# Non-contact heart vibration measurement using computer vision-based seismocardiography

**DOI:** 10.1038/s41598-023-38607-7

**Published:** 2023-07-21

**Authors:** Mohammad Muntasir Rahman, Jadyn Cook, Amirtahà Taebi

**Affiliations:** Department of Agricultural and Biological Engineering, Mississippi State University, Mississippi, 39762 USA

**Keywords:** Biomedical engineering, Computer science, Electrical and electronic engineering

## Abstract

Seismocardiography (SCG) is the noninvasive measurement of local vibrations of the chest wall produced by the mechanical activity of the heart and has shown promise in providing clinical information for certain cardiovascular diseases including heart failure and ischemia. Conventionally, SCG signals are recorded by placing an accelerometer on the chest. In this paper, we propose a novel contactless SCG measurement method to extract them from chest videos recorded by a smartphone. Our pipeline consists of computer vision methods including the Lucas–Kanade template tracking to track an artificial target attached to the chest, and then estimate the SCG signals from the tracked displacements. We evaluated our pipeline on 14 healthy subjects by comparing the vision-based SCG$^\mathrm{{v}}$ estimations with the gold-standard SCG$^\mathrm{{g}}$ measured simultaneously using accelerometers attached to the chest. The similarity between SCG$^\mathrm{{g}}$ and SCG$^\mathrm{{v}}$ was measured in the time and frequency domains using the Pearson correlation coefficient, a similarity index based on dynamic time warping (DTW), and wavelet coherence. The average DTW-based similarity index between the signals was 0.94 and 0.95 in the right-to-left and head-to-foot directions, respectively. Furthermore, SCG$^\mathrm{{v}}$ signals were utilized to estimate the heart rate, and these results were compared to the gold-standard heart rate obtained from ECG signals. The findings indicated a good agreement between the estimated heart rate values and the gold-standard measurements (bias = 0.649 beats/min). In conclusion, this work shows promise in developing a low-cost and widely available method for remote monitoring of cardiovascular activity using smartphone videos.

## Introduction

In the United States, cardiovascular diseases (CVDs) are the number one leading cause of death, and they remained the top cause of death during the COVID-19 pandemic, accounting for 20.1% of all fatalities between March 2020 and October 2021^1^. According to the American Heart Association, CVDs impose an enormous health and economic burden in the United States and worldwide^2^. This mortality rate and economic burden can be reduced by improving diagnostic methods and making them more accessible to achieve earlier detection of cardiac abnormalities. In that regard, routine monitoring of cardiac activity can increase the possibility of early diagnosis of CVDs. Current cardiac activity monitoring methods include both invasive (such as pulmonary artery catheterization^3^) and non-invasive techniques. The invasive techniques are usually performed in clinical facilities limiting their utility for routine remote monitoring of the patients. On the other hand, non-invasive methods such as electrocardiography (ECG) have been widely used to develop remote monitoring systems that can be used outside of healthcare facilities. Recent studies on seismocardiography (SCG), another non-invasive technique that records cardiovascular vibrations on the chest surface, suggested their potential in accurately estimating clinically significant parameters^4^. These vibrations can be measured in dorsoventral, right-to-left, and head-to-foot directions, and are caused by heart mechanical activities, such as mitral and aortic valves opening and closing, isovolumetric contraction, ejection, and rapid filling of left ventricle^4–6^. Since SCG measures the mechanical activity of the heart, it can provide complementary diagnostic information to other modalities such as ECG and pulse oximetry that evaluate the electrical activity of the heart and the blood oxygen level^4,7,8^. As a result, several studies demonstrated that SCG signals contain information that can be utilized to precisely investigate cardiac activity such as the timing of the opening and closing of the aortic and mitral valves^9,10^. Also, SCG signals have shown promise in detecting and monitoring a variety of cardiovascular diseases, such as coronary heart disease, myocardial infarction, ischemia, and hemorrhage^11–17^.

SCG signals are conventionally measured using accelerometers attached to the chest surface. With the advancement of sensors and technology, the concept of non-contact monitoring of cardiac activity has received increasing attention due to its numerous advantages over traditional techniques. Previous studies utilized various technologies including infrared sensors^18^, radars^19^, or even WiFi devices^20,21^ for non-contact vital sign monitoring. While these methods have demonstrated success in laboratory settings, their practical implementation for cardiac monitoring outside of laboratories or clinical facilities is impeded by their bulky or costly hardware. In recent years, with the proliferation of digital communication devices such as smartphones and laptops, the research community has become increasingly interested in computer vision-based remote cardiac activity monitoring^22,23^. Vision-based health assessment offers distinct benefits such as being non-contact, non-invasive, and easy to use. These methods have been used in different applications, including heart and respiration rate monitoring, heart rate variability assessment, and blood oxygen saturation evaluation^24–28^. However, to the best of our knowledge, the majority of computer vision-based cardiac monitoring studies mainly focused on measuring heart rate and did not extract low-amplitude high-frequency events from individual heartbeats relevant to SCG^29–31^.

The aim of this study is to determine the feasibility of using a smartphone camera and computer vision to acquire a seismocardiogram. Thus, our primary objective is to estimate SCG signals in the right-to-left and head-to-foot directions from video using computer vision techniques, which could provide a novel method to investigate the cardiac-induced vibrations on the chest, and eventually build the foundation for developing an accessible low-cost cardiac monitor. Similar to capturing data from multiple accelerometers on the chest surface, we used a smartphone camera to track multiple target regions on the chest surface to measure the acceleration signals induced by the heart activity. The main advantage of the proposed method in this study is that it uses a smartphone camera to acquire data, which is relatively easy to set up and may be deployed immediately in emergency situations. Vision-based approaches also offer the ability to monitor multi-point measurements with a single camera sensor.

## Materials and methods

### Study population

The study protocol was approved by the institutional review board (IRB) of Mississippi State University and all research was performed in accordance with the guidelines and regulations described in the IRB protocol. A total of 14 subjects with no history of CVDs and from diverse backgrounds were recruited (50% White, 21.5% Black, 21.5% Asian, and 7% mixed). Table 1 shows the study population’s age, height, weight, and body mass index (BMI). The subjects signed an informed consent form and completed a short survey about their health conditions prior to the study.

**Table 1:** Overview of the study population characteristics.

Subject	Mean ± SD
Age (year)	23.50 ± 5.16
Height (cm)	170.80 ± 9.35
Weight (kg)	70.07 ± 13.97
BMI (kg/m$^2$)	23.93 ± 4.07
Number of subjects	14 (4 females)

### Experimental protocol

**Figure 1:**
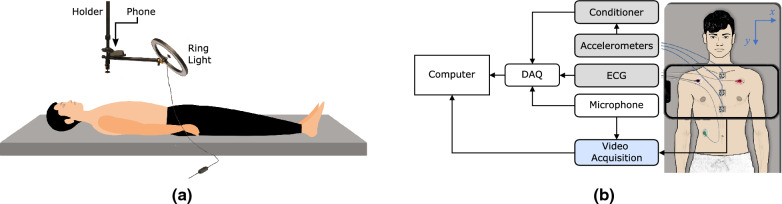
Experimental setup: (**a**) subjects were asked to lay down in a supine position. A smartphone was held by a phone holder to videotape the subject’s chest. (**b**) The arrangement of the signal and video acquisition systems.

All subjects were instructed to lay supine on a bed without additional body movements (Fig. 1a). To minimize the respiration noise, data was acquired during a 15-s breath-hold at the end of inhalation followed by another 15-s breath-hold at the end of exhalation.

#### Vision-based SCG (SCG$\mathrm{^{v}}$)

A smartphone (iPhone 13 Pro, Apple Inc, Cupertino, CA) was used to videotape the upper chest of the subjects with an acquisition speed of 60 frames per second (fps) and resolution of $3840 \times 2160$. A phone holder was used to keep the smartphone stationary with the back camera facing the subject’s chest. To minimize the phone vibrations, a Bluetooth remote control was used to start and stop the recording. We placed three texture-patterned QR codes on the chest surface as a high-contrast artificial region of interest to be tracked by our computer vision algorithm, since the sufficient intensity variation in the target region provides reliable recognition and matching in target tracking^32^.

#### Gold-standard SCG signals (SCG$\mathrm{^{g}}$)

In order to validate the vision-based SCG signals, a triaxial accelerometer (356A32, PCB Piezotronics, Depew, NY) was placed underneath each QR code. The accelerometers were then attached to three locations on the sternum including the manubrium, the fourth costal notch, and the xiphoid process. A signal conditioner (482C, PCB Piezotronics, Depew, NY) was used to amplify the accelerometer outputs with a gain factor of 100. The amplified signals (i.e., SCG$^\mathrm{{g}}$) were then recorded using a data acquisition system, DAQ, (416, iWorx Systems, Inc., Dover, NH) with a sampling frequency of 5000 Hz.

The accelerometer outputs and the chest videos were recorded simultaneously using two independent systems (i.e., the DAQ and phone) with different sampling frequencies. Therefore, to synchronize the SCG$^\mathrm{{g}}$ and SCG$^\mathrm{{v}}$ signals in the later stages of the data analysis, a microphone was connected to both of these systems and was tapped at the beginning and end of each recording. These taps were then identified in the audio of the video and the sound signal recorded by the DAQ to synchronize the signals. In addition, ECG signals were recorded in each experiment (iWire-B3G, iWorx Systems, Inc., Dover, NH) and were used for SCG segmentation as will be described later. Figure 1b shows the sensor locations and the direction of *x* and *y*-axes in this study.

### Vision-based SCG measurement method

**Figure 2:**
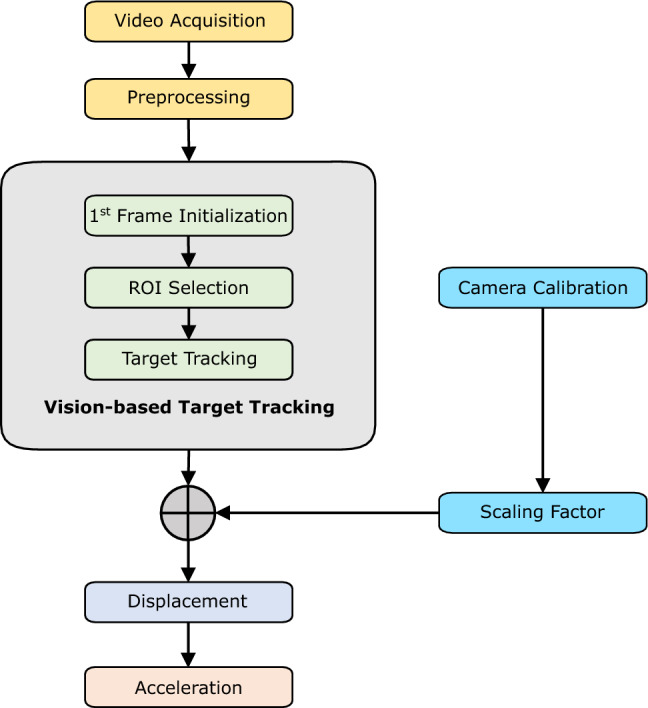
Vision-based SCG estimation pipeline. The pipeline consists of four main steps: preprcessing, target tracking, camera calibration, and acceleration calculation.

Our vision-based SCG signal measurement is based on tracking artificial targets (i.e., QR codes) on the chest surface. As shown in Fig. 2, the videos were pre-processed after the video acquisition. We then employed a target-tracking algorithm to measure the displacement of the target regions. Camera calibration was then performed to convert the displacements from pixels to millimeters. Finally, we computed the acceleration signal from the displacements. Overall, the pipeline consists of four steps: (1) preprocessing; (2) vision-based tracking and displacement calculation; (3) camera calibration; and (4) acceleration calculation. Each step is described below.

#### Preprocessing

To calculate the SCG signals in the *x* and *y* directions as depicted in Fig. 1b, the videos were rotated, if needed, on a case-by-case basis such that the QR codes are aligned with the camera’s *x*- and *y*-axes. This step was done for each QR code separately to ensure that the *x* and *y*-axes of the video and accelerometers are as aligned as possible.

#### Vision-based tracking and displacement calculation

Computer vision methods are widely used for object tracking and displacement measurement in videos. Vision-based displacement estimation methods, such as tracking techniques and optical flow compute relative displacement by correlating successive image frames in a video. Template matching is the most prevalent tracking technique, which works by searching for the image regions that are most similar to a reference template. In the present study, we employed an optical flow-based template tracking method to extract the displacement information of the target regions on the chest. More specifically, we used the Lucas–Kanade template tracking algorithm^33^ to measure the displacement of the QR codes attached on the chest. In this regard, we adapted the unified approach proposed in^34^ for the Lucas–Kanade method for QR code tracking, with the goal of minimizing the sum of the squared error between two images, i.e., the QR code itself and a video frame in which the QR code is being searched for. The minimization is done based on warping the video frame onto the coordinate frame of the QR code. Since this optimization is nonlinear, it is performed iteratively solving for increments to update previously known warping parameters.

Given a chest video sequence *I*(*x*), let $I_n({x})$ represents the $n$th video frame in which we looked for a region to match the QR code template, where ${x}=(x,y)^T$ is a column vector that contains the pixel coordinates and $n=1,2,\dotsc ,N$ represents the frame number. Template *QR*(*x*) is the target region defined from the first frame $I_1({x})$ of the chest video. The motion of the QR code was then calculated by mapping the *QR*(*x*) to the successive frames $I_n({x})$. The mapping was done using a warp function *W*(*x*; *u*), which was parameterized by vector ${u}=(u_1,\dotsc ,u_k)^T$. The warp function *W*(*x*; *u*) transforms the pixel *x* in the coordinate frame of the *QR*(*x*) to the sub-pixel position *W*(*x*; *u*) in the coordinate frame of the video frame $I_n({x})$. Assuming that the QR code is flat, parallel to the camera plane, and not rotated, the warp function *W*(*x*; *u*) can be then computed using the following 2D image transformation, where the parameters ${u}=(u_1,u_2)^T$ is the motion vector.

1 $$\begin{aligned} {{W(x;u)} = \left( \begin{array}{cc} x+u_1 \\ y+u_2 \end{array}\right) } \end{aligned}$$

The goal of the tracking algorithm is now to find optimal parameters *u* such that the warped image $I_n({W(x;u)})$ and the *QR*(*x*) are perfectly aligned. The region that matches the QR code in a new frame is determined by optimizing the parameters *u* to minimize the sum of squared difference error between $I_n({W(x;u)})$ and *QR*(*x*) as

2 $$\begin{aligned} {E=\sum _{{x}\in QR}\left[ I_n({W(x;u)})-QR({x})\right] ^2} \end{aligned}$$

where the error between the intensity of each pixel *x* of the QR code and its corresponding pixel in the video frame $I_n({W(x;u)})$ is measured. In order to compute $I_n({W(x;u)})$, it is necessary to interpolate the image $I_n$ at the sub-pixel positions *W*(*x*; *u*). The minimization of the expression in (2) is a non-linear optimization problem, which is solved by iteratively updating the parameters *u* using the increments ${\Delta u}$ assuming that the current estimation of *u* is known. Consequently, (2) can be rewritten as

3 $$\begin{aligned} E=\sum _{{x}\in QR}\left[ I_n({W(x;u+\Delta u)})-QR({x})\right] ^2 \end{aligned}$$

and parameters *u* are iteratively updated as

4 $$\begin{aligned} {{u\leftarrow u+\Delta u}} \end{aligned}$$

The non-linear (3) can be linearized by employing the first-order Taylor approximation of $I_n({W(x;u+\Delta u)})$ as

5 $$\begin{aligned} {I_n({W(x;u+\Delta u)}) = I_n({W(x;u)})+{\nabla I_n}\frac{\partial {W}}{\partial {u}}\Delta {u}} \end{aligned}$$

where ${\nabla I_n} = \left( {\partial I_n}/{\partial x}, {\partial I_n}/{\partial y} \right)$ represents the *gradient* of the video frame $I_n$ evaluated at the current estimation of the warp *W*(*x*; *u*), and ${\partial {W}}/{\partial {u}}$ is the *Jacobian* of the warp function *W*(*x*; *u*). The first-order approximation of $I_n({W(x;u+\Delta u)})$ from (5) can be substituted in (3) as

6 $$\begin{aligned} {E=\sum _{{x}\in QR}\left[ I_n({W(x;u)})+{\nabla I_n}\frac{\partial {W}}{\partial {u}}\Delta {u}-QR({x})\right] ^2} \end{aligned}$$

Minimization of the error function in (6) is a least-squares problem that can be solved by taking partial derivative of the error function *E* with respect to ${\Delta u}$ as

7 $$\begin{aligned} \frac{\partial E}{\partial {\Delta u}}=&2\sum _{{x}\in QR}\left[ {\nabla I_n}\frac{\partial {W}}{\partial {u}} \right] ^T [ I_n({W(x;u)}) +{\nabla I_n}\frac{\partial {W}}{\partial {u}}\Delta {u}-QR({x}) ] \end{aligned}$$

and then set ${\partial E}/{\partial {\Delta u}}=0$, which results in

8 $$\begin{aligned} {{\Delta u} = H^{-1}\sum _{{x}\in QR}\left[ {\nabla I_n}\frac{\partial {W}}{\partial {u}} \right] ^T\left[ QR({x})-I_n({W(x;u)}) \right] } \end{aligned}$$

where *H* represents the $n\times n$ Hessian matrix from the Gauss–Newton approximation and is defined as

9 $$\begin{aligned} {H=\sum _{{x}\in QR}\left[ {\nabla I_n}\frac{\partial {W}}{\partial {u}} \right] ^T\left[ {\nabla I_n}\frac{\partial {W}}{\partial {u}} \right] } \end{aligned}$$

The steps in (8) and (4) are iterated until the convergence of *u* (i.e., $\Vert {\Delta u}\Vert \le \epsilon$, where $\epsilon$ is a threshold). We also defined a limit for the maximum number of iterations $i^{max}$ to reduce the computational cost. The pseudo-code of our vision-based tracking is shown in Algorithm 1.
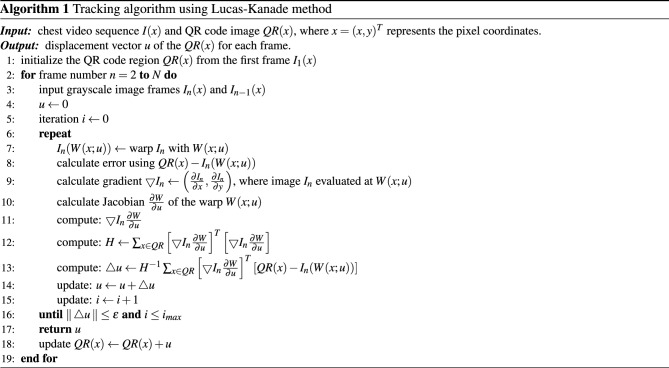


#### Camera calibration

**Figure 3:**
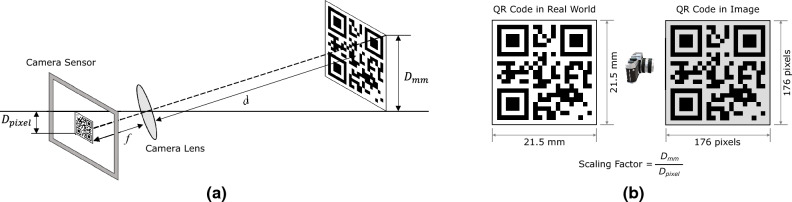
Camera calibration and calculating the scaling factor. (**a**) Pinhole camera model, (**b**) schematic diagram of scaling factor calculation for converting the pixel unit to length unit (mm). $D_{mm}$ and $D_{pixel}$ are the physical distance of two points in millimeters and the distance of the same points in the image pixel, respectively.

To convert the displacement obtained by the target tracking algorithm from pixel to millimeter, a geometric relationship between the 2D image coordinate system and the world coordinate system needs to be established. This was done by the camera calibration process. When calibrating the camera, we attempted to align the camera coordinate system with the world coordinate system. The scaling factor is one of the most common ways to calibrate a camera for measuring displacement. When the optical axis of the camera is perpendicular to the object plane, all points on that plane can be assumed to have the same depth of field and can be similarly scaled down to the image plane. Generally, the scaling factor can be computed using the following two methods^35^. First, $SF_1=D_{mm}/D_{pixel}$ computes the ratio of the physical dimension of the object surface in millimeters or inches in the world coordinate system to the corresponding dimension in pixels in the image frame, where $D_{mm}$ is the physical length of the artificial target in millimeter, and $D_{pixel}$ is the corresponding pixel length (Fig. 3a). Second, $SF_2=d\times p/f$ is based on the ratio of the distance between the camera and the target object to the focal length of the camera. Here, *d* is the distance from the camera to the object surface, *f* is the focal length, and *p* is the unit length of the camera sensor ($\mu$m/pixel). In this study, since the image plane was parallel to the motion of the target region, we employed the $SF_1$ scaling factor. For the unit conversion from image pixels to millimeters, we first measured the actual physical size of the QR code, and then calculated the pixel dimension of the same QR code on the image, as illustrated in Fig. 3b. Using these two values, we then determined the scaling factor.

#### Acceleration calculation

After calculating the displacement signal and converting it into millimeters, the acceleration signal (i.e., SCG$^\mathrm{{v}}$) was derived by calculating the second derivative of the displacement signal.

### Validation of vision-based SCG estimations

We validated our computer vision pipeline by comparing the SCG estimations with the gold-standard signals in time and frequency domains.

**Figure 4:**
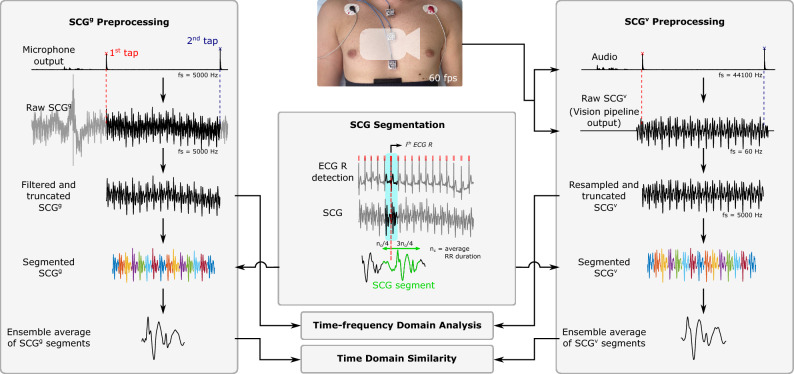
Overview of the gold-standard SCG$^\mathrm{{g}}$ and vision-based SCG$^\mathrm{{v}}$ preprocessing. The output of the microphone was recorded simultaneously by both the camera and DAQ to synchronize the signals. The raw SCG$^\mathrm{{g}}$ was bandpass filtered and the SCG$^\mathrm{{v}}$ was resampled to 5000 Hz. Both signals were then segmented using the ECG R waves, and the ensemble averages of their segments were computed. The time domain similarity analysis was performed between the ensemble averages of the SCG segments. For the time–frequency analysis, the whole preprocessed signals were used.

#### Signal preprocessing

To compare the SCG signals recorded by the accelerometers with those estimated from the chest video, both SCG$^\mathrm{{g}}$ and SCG$^\mathrm{{v}}$ signals were first preprocessed. Figure 4 shows an overview of the signal processing pipeline in this study. The accelerometer outputs were filtered using a band-pass filter with cutoff frequencies of 1 and 30 Hz. This was done because the SCG estimations from the video could capture vibrations up to half of the camera acquisition speed (i.e., 60 fps). Additionally, the vision-based SCG signals were resampled using linear interpolation to 5000 Hz (i.e., the sampling frequency of the gold-standard signals). The raw SCG$^\mathrm{{v}}$ with the sampling frequency of 60 Hz was missing some waveform features such as the curvature between two consecutive sample points. The resampling step was done to obtain a smoother SCG$^\mathrm{{v}}$ by reconstructing the missed features and had a minimal effect on the signals’ contents in time and frequency domains. The resampling step also included the employment of an FIR antialiasing lowpass filter and accounted for the delay introduced by the filter. SCG signals were then segmented into cardiac cycles using the ECG R waves identified using Pan–Tompkin algorithm^36,37^. First, the average cardiac cycle duration in terms of the number of sample points, $n_c$, was calculated for each subject using the ECG RR intervals. Then, SCG segments were defined as $SCG(n_i-n_c/4:n_i+3 \times n_c/4)$, where $n_i$ denotes the time index of the *i*th ECG R wave.

#### Time domain similarity

The ensemble averages of the SCG segments, $\overline{\text {SCG}}^{g}$ and $\overline{\text {SCG}}^{v}$, were calculated and used for the time domain similarity analysis. The ensemble averaging helped remove the beat-to-beat variability and noise. The Pearson correlation coefficient between $\overline{\text {SCG}}^{g}$ and $\overline{\text {SCG}}^{v}$ was calculated for the signals recorded from all three locations. This coefficient represents how closely the two signals are correlated. However, any time lags between two similar signals may lead to a low correlation coefficient. Therefore, in this study, we also used dynamic time warping (DTW) to assess the similarity of the signals^38^. For this purpose, Euclidean distances between the ensemble average of the vision-based and gold-standard SCG segments, $D(\overline{SCG}\mathrm{^{g}},\overline{SCG}\mathrm{^{v}})$, were first calculated using DTW while the warping path was restricted to be within a 5% distance threshold from the straight-line fit. The similarity index, *S*, between the ensemble averages was then defined and calculated as

10a $$\begin{aligned} S_x(\overline{SCG}_x^g,\overline{SCG}_x^v) = \frac{M(\overline{SCG}_x^g) - D(\overline{SCG}_x^g,\overline{SCG}_x^v)}{M(\overline{SCG}_x^g)} \end{aligned}$$

10b $$\begin{aligned} S_y(\overline{SCG}_y^g,\overline{SCG}_y^v) = \frac{M(\overline{SCG}_y^g) - D(\overline{SCG}_y^g,\overline{SCG}_y^v)}{M(\overline{SCG}_y^g)} \end{aligned}$$

where $M(\overline{SCG}\mathrm{^{g}})$ is the maximum of the absolute value of the gold-standard SCG$^\mathrm{{g}}$ segments multiplied by the length of one segment, i.e., $n_c$. This normalized similarity index is always in the range of $\left[ 0,1 \right]$.

#### Wavelet coherence analysis

The correlation between the gold-standard and vision-based SCG signals in the time–frequency plane was calculated using magnitude-squared wavelet coherence ($C_{g,v}$) as^39^

11 $$\begin{aligned} C_{g,v}(a,b)=\frac{\left| \mathscr{S}(C_g^{*}(a,b) C_v(a,b)) \right| ^2}{ \mathscr{S}(\left| C_g(a,b) \right| ^2) . \mathscr{S}(\left| C_v(a,b) \right| ^2)} \end{aligned}$$

where $C_g(a,b)$ and $C_v(a,b)$ are the continuous wavelet transforms of the signals SCG$^\mathrm{{g}}$ and SCG$^\mathrm{{v}}$ at scales *a* and positions *b*. $\mathscr{S}(\cdot )$ is a smoothing function in time and scale, and the superscript $*$ is the complex conjugate operator. The coherence was calculated using the analytic Morlet wavelet since previous work showed that Morlet wavelet estimates the frequency content of SCG signals more accurately than other mother functions^40^. The correlations were calculated for the duration of the recordings and in the range of 0–30 Hz. $C_{g,v}$ is always in the range of $\left[ 0,1 \right]$, with $C_{g,v}=1$ indicating the highest correlation between the two signals in the frequency domain.

#### Heart rate estimation

The heart rate (HR) of subjects was estimated using SCG$^\mathrm{{v}}$. To evaluate the accuracy of these estimations, they were compared with the gold-standard HR values obtained from the ECG signals ($HR_{ECG}$). $HR_{ECG}$ was calculated in beats per minute (bpm) by determining the temporal indices of the ECG R peaks using the Pan–Tompkin algorithm, and then substituting them in Eq. (12):

12 $$\begin{aligned} { HR_{ECG}^i = \frac{1}{t(n_{i+1})-t(n_i)} \times 60 } \end{aligned}$$

where, $n_i$ represents the time index of the *i*th ECG R peak. To estimate the HR from SCG$^\mathrm{{v}}$ ($HR_{SCG}$), we developed an algorithm based on bandpass filtering of the signals and finding the peaks of the filtered signals (Algorithm 2).
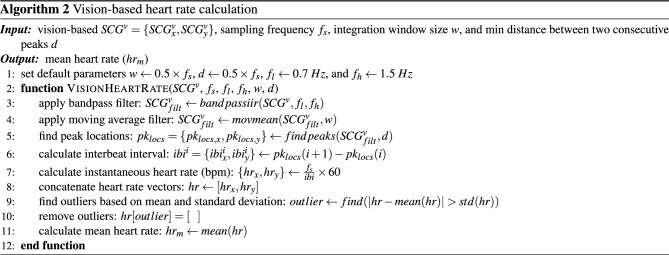


## Results

### Vision-based SCG estimations

**Figure 5:**
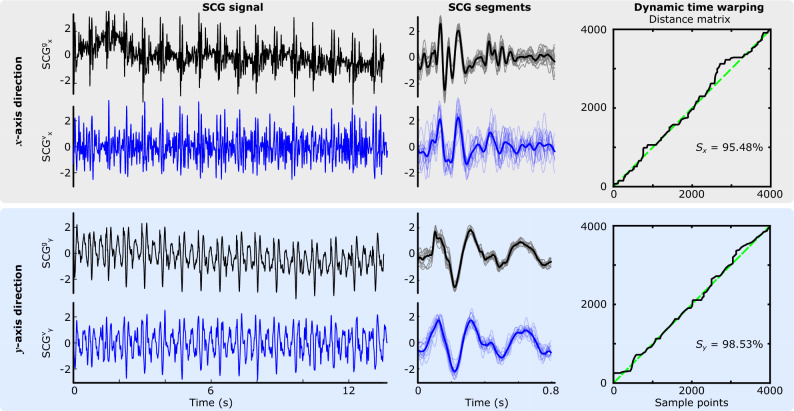
Gold-standard SCG$^\mathrm{{g}}$ (black) and vision-based SCG$^\mathrm{{v}}$ (blue) signals in *x* (right-to-left) and *y* (head-to-foot) directions (top and bottom panels, respectively) obtained from a male participant during breath-hold at the end of inhalation (subject 9, the middle accelerometer and QR code). SCG segments are shown in the middle panel. The bold black and blue waveforms represent the ensemble average of the segments. The distance matrix of dynamic time warping analysis, the warping path (black line), the straight-line fit (green dashed line), and the similarity index *S* using (10) are shown at right.

Our goal was to understand if the SCG signals can be extracted from the chest videos recorded by a smartphone. We estimated the SCG signals in both *x* and *y* directions using our computer vision pipeline for all three sensor locations. Figure 5 shows the vision-based SCG and the gold-standard accelerometer data for one of the male subjects (subject 9, during breath-hold at the end of inhalation). The SCG signals were then segmented into cardiac cycles using ECG R waves as described in “Signal preprocessing”. The SCG segments and their ensemble averages are shown in the middle panel of Fig. 5. Qualitative comparison of the ensemble average of the SCG$^\mathrm{{g}}$ and SCG$^\mathrm{{v}}$ segments indicated that our vision-based pipeline was able to capture the main features of the $\mathrm{SCG_{x}}$ and $\mathrm{SCG_{y}}$. However, the intragroup variability of the SCG$^\mathrm{{v}}$ segments was more than the SCG$^\mathrm{{g}}$ segments, especially in the *x* direction. Similar results were obtained for other subjects.

### Signal similarity analysis

We analyzed the vision-based estimations of $\mathrm{SCG_{x}}$ (right-to-left direction) and $\mathrm{SCG_{y}}$ (head-to-foot direction) in time and frequency domains to quantitatively determine if they are comparable to those obtained from the accelerometers attached to the chest skin. For this purpose, we calculated the similarity between the $\overline{\text {SCG}}^{g}$ and $\overline{\text {SCG}}^{v}$ of all the subjects recruited in this study. One of the chest videos of a female subject (subject 4, breath-hold at the end of exhalation) was not saved properly during the data acquisition step and therefore was excluded from further analysis. Considering that for each subject, the $\mathrm{SCG_{x}}$ and $\mathrm{SCG_{y}}$ signals were measured from three locations on the chest, and during breath-hold at the end of inhalation and exhalation, there were a total of 162 pairs of $\overline{\text {SCG}}^{g}$ and $\overline{\text {SCG}}^{v}$ for the validation of the proposed computer vision pipeline.

#### Time domain similarity

**Figure 6:**
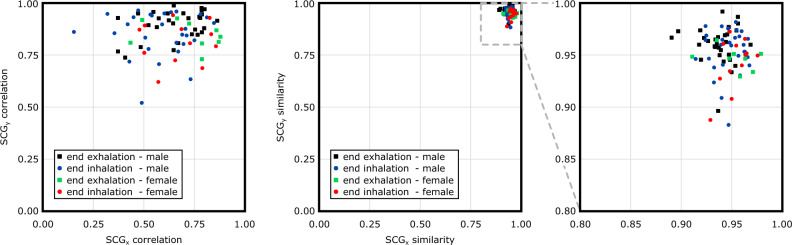
Correlation and similarity index between the gold-standard SCG$^\mathrm{{g}}$ and vision-based SCG$^\mathrm{{v}}$. Each data point represents the comparison between an accelerometer data and the SCG estimated from the corresponding QR code. Three $\mathrm{SCG_{x}}$ pairs of subject 11 had a negative correlation coefficient and were not shown in the left plot.

The temporal similarity of the ensemble averages of the SCG$^\mathrm{{g}}$ and SCG$^\mathrm{{v}}$ segments was evaluated by computing the correlation coefficient between the signals (the left panel of Fig. 6). In this graph, the *x* coordinate of each data point represents the correlation coefficient between the $\overline{\text {SCG}}^\text {g}_x$ and $\overline{\text {SCG}}^\text {v}_x$. Similarly, the correlation coefficient between the $\overline{\text {SCG}}^\text {g}_y$ and $\overline{\text {SCG}}^\text {v}_y$ is shown on the *y*-axis. Therefore, when both $\text {SCG}_x^\text {v}$ and $\text {SCG}_y^\text {v}$ signals obtained from a QR code exhibit high correlations with the gold-standard $\text {SCG}^\text {g}$, the corresponding point on the plot will appear in the top right corner. Conversely, if both $\text {SCG}^\text {v}$ signals in the *x* and *y* directions demonstrate a low correlation with $\text {SCG}^\text {g}$, the corresponding point will fall into the bottom left corner of the plot. Results showed that the correlation between the gold-standard and vision-based $\mathrm{SCG_{y}}$ pairs was higher than the correlation between $\mathrm{SCG_{x}}$ pairs. In other words, while all $\mathrm{SCG_{y}}$ pairs had a correlation of 0.5 or larger (the upper half of the graph), $\mathrm{SCG_{x}}$ pairs presented a lower correlation with many pairs falling in the left half of the graph, i.e., a correlation coefficient of less than 0.5 which included three pairs (all from subject 11) that had a negative correlation (not shown in Fig. 6). More specifically, the average correlation between $SCG_{y}$ pairs was 0.86 with a maximum of 0.99. On the other hand, the average correlation between the $\mathrm{SCG_{x}}$ pairs was 0.60 with a maximum of 0.88.

The similarity of the signals in the time domain was also investigated using DTW. The right panel of Fig. 5 shows the distance matrices in the *x* and *y* directions for the middle accelerometer and QR code of subject 9 (breath-hold at the end of inhalation). The Euclidean distance between the gold-standard and vision-based signals was calculated from the distance matrices and used to calculate the similarity indices $S_x$ and $S_y$. The similarity indices for other subjects were calculated similarly and reported in the middle panel of Fig. 6). The *x* and *y* coordinates of the data points in this graph represent the $S_x$ and $S_y$, respectively. High similarity between $\text {SCG}^\text {v}$ signals from a QR code and the gold-standard $\text {SCG}^\text {g}$ result in points in the top right corner of the plot. Conversely, low correlations place the corresponding points in the bottom left corner. Results showed that the similarity of the ensemble averages in both the *x* and *y* directions was above 0.85. The average, maximum, and minimum similarity between the $\mathrm{SCG_{x}}$ pairs were 0.94, 0.98, and 0.89, respectively. High similarity values were also obtained for the $\mathrm{SCG_{y}}$ pairs (0.95, 0.99, and 0.88, respectively). No meaningful differences between similarity indices of different groups of data (e.g., male vs. female, or end-inhalation vs. end-exhalation) were observed.

#### Wavelet coherence analysis

**Figure 7:**
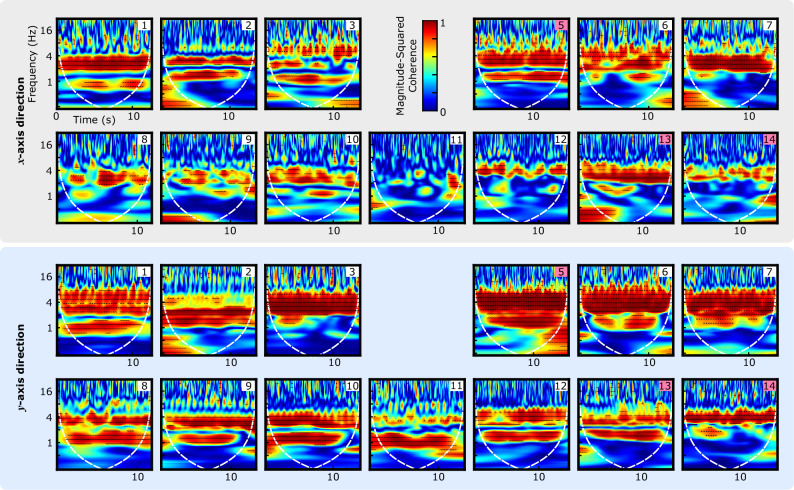
Wavelet coherence between the gold-standard SCG$^\mathrm{{g}}$ and vision-based SCG$^\mathrm{{v}}$ signals calculated for the breath-hold experiment at the end of exhalation. The white dashed line shows the cone of influence. Subject numbers are indicated on the top right corner of each plot. Pink numbers correspond to female subjects. For this experiment, the video from subject 4 (female) was not saved properly and, therefore, is missing.

Figure 7 shows the magnitude-squared wavelet coherence plots for the middle location of all the subjects during breath-hold at the end of exhalation. The white dashed line is the cone of influence that distinguishes the areas of the scalogram that may be affected by edge effect artifacts (i.e., outside the dashed line) from those that represent accurate time–frequency information (i.e., inside the dashed line). Results showed that the spectrotemporal correlation of the $\mathrm{SCG_{y}}$ signals was higher than their corresponding $\mathrm{SCG_{x}}$ pairs. Most pairs had a high correlation in the lower frequency bands of 1–5 Hz. The signal pairs of some subjects also had a high correlation in the middle-frequency bands. For example, the wavelet coherence between $\text {SCG}_y^\text {g}$ and $\text {SCG}_y^\text {v}$ of subject 6 was above 0.9 in the frequency band of 4–10 Hz. In the higher frequency bands (i.e., 15–30 Hz), the signal correlations were lower than those in the lower frequency bands. However, the signals had a correlation of 0.6–0.9 during the timing of the heart sounds (i.e., SCG1 and SCG2 based on the definition in a previous study^4^).

#### Heart rate agreement analysis

**Figure 8:**
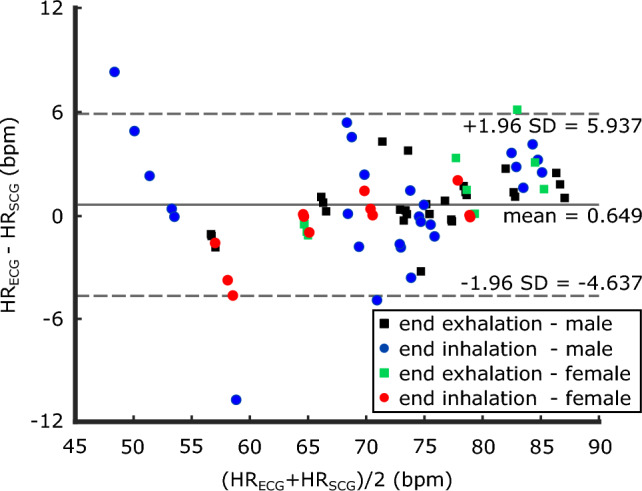
Bland–Altman plot for heart rates estimated from ECG ($\mathrm{HR_{ECG}}$) and SCG$^\mathrm{{v}}$ ($\mathrm{HR_{SCG}}$) derived from the aggregated data of all subjects (male, female; breath-hold at the end of exhalation, and end of inhalation), except subject 11. Mean bias (solid line), and upper and lower limits of agreement, i.e., mean ± 1.96 SD (dashed lines) are shown.

Bland–Altman plot was used to determine the level of agreement between the gold-standard $\mathrm{HR_{ECG}}$ and $\mathrm{HR_{SCG}}$ estimated from the vision-based SCG signals (Fig. 8). Due to the lowest similarity observed between the SCG$^\mathrm{{v}}$ signals of subject 11 and the gold-standard SCG$^\mathrm{{g}}$, we excluded this particular subject from the HR analysis. Bias or the mean difference of HR estimations between ECG and SCG$^\mathrm{{v}}$ was 0.649 bpm. The lower and upper limits of agreement ranged from − 4.637 to 5.937 bpm.

## Discussion

This work shows that cardiac-induced vibrations can be extracted from the videos recorded of the chest. We validated our vision-based pipeline by evaluating the similarity between the SCG$^\mathrm{{v}}$ and the gold-standard SCG$^\mathrm{{g}}$ recorded by the accelerometers attached to the chest skin. Time domain results demonstrated a high DTW similarity index, but a lower correlation coefficient, especially between the $\text {SCG}^\text {g}_x$ and $\text {SCG}^\text {v}_x$. This could be partly because the SCG$^\mathrm{{g}}$ and SCG$^\mathrm{{v}}$ signals were acquired simultaneously using two independent systems with different sampling frequencies. Although we developed strategies to synchronize the output of these systems, there was still a time lag between the vision-based and gold-standard signals in each experiment. The average lag between $\mathrm{SCG_{x}}$ pairs and $\mathrm{SCG_{y}}$ pairs were $52\pm 112$ ms and $41\pm 104$ ms, respectively. The DTW algorithm accounted for this lag by stretching the SCG$^\mathrm{{v}}$ signal onto the SCG$^\mathrm{{g}}$ such that the Euclidean distances between the corresponding points are smallest. On the other hand, the presence of this time lag resulted in lower correlation coefficients. Therefore, the higher DTW-based similarity indices may provide a more accurate evaluation of the signal similarities and the performance of our computer vision pipeline.

The SCG$^\mathrm{{v}}$ signals were employed to estimate the subjects’ HR. In general, there was a good agreement observed between the gold-standard HR measurements and the HR estimations derived from SCG$^\mathrm{{v}}$ signals. However, it is important to mention that a basic algorithm based on bandpass filtering of signals between 0.7 and 1.5 Hz was utilized for HR estimation from the SCG signals. This simplified approach was chosen as the focus of this study did not involve the development of an advanced HR estimation algorithm. Consequently, it is possible that the relatively lower agreements between some SCG$^\mathrm{{v}}$-based HR estimations and the gold standard were influenced, as exemplified by the two outliers at the end of inhalation for male subjects (shown by blue circles above and below the limits of agreement in Fig. 8). More specifically, HR values lower than 60 bpm exhibited a wider dispersion in the Bland–Altman plot. Moreover, our SCG$^\mathrm{{v}}$ and HR estimation algorithm tended to slightly underestimate HR for the values above 81 bpm. Future work can focus on enhancing the HR estimation algorithm to obtain a more comprehensive understanding of the potential of SCG$^\mathrm{{v}}$ signals in estimating HR and cardiac time intervals.

When a triaxial accelerometer is placed on the chest, SCG components are measured in all three *x* (right-to-left), *y* (head-to-foot), and *z* (dorsoventral) axes, each displaying a specific pattern. However, our vision-based pipeline could only estimate the SCG signals along the *x* and *y* directions. In the literature, most SCG studies including those that used a contactless measurement method, focused on the dorsoventral component of the SCG^7,10^. Although the dorsoventral component cannot be currently estimated by our method, it is plausible that additional and complementary diagnostic information can be extracted from the analysis of the right-to-left and head-to-foot SCG components^7^. For example, Shandhi et al. reported that the changes in the pulmonary artery mean pressure and the pulmonary capillary wedge pressure can be better estimated from the changes in the SCG signals along the *x*-axis^14^. In addition, in this study, the videos were captured with an acquisition speed of 60 fps, allowing us to estimate the SCG features up to 30 Hz, i.e., the infrasonic SCG features^41^. Since SCG signals may contain clinically relevant information for specific CVDs in the higher frequency ranges^42^, we will develop an experimental setup and pipeline to estimate the higher frequency features as well as the SCG dorsoventral components in our future efforts.

**Figure 9:**
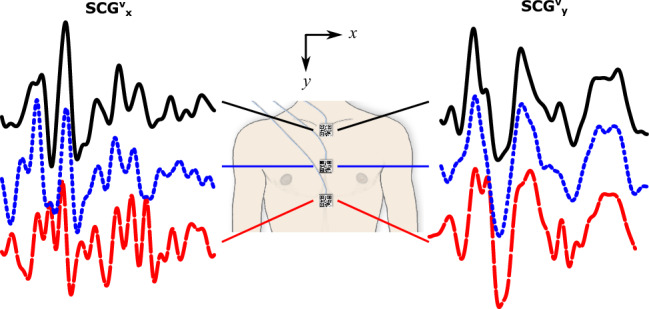
Simultaneous vision-based SCG estimations from multiple chest locations (results for subject 9, breath-hold at the end of inhalation).

It is well known that the SCG signals vary with their measurement locations on the chest^4^. The conventional methods to investigate SCG variability includes employing an array of accelerometers attached to the chest. For example, Azad et al. used 32 accelerometers in their array between the right and left anterior axillary and between the 2nd and 5th intercostal spaces^43^. Although increasing the number of sensors in such arrays can provide a better picture of the spatial distribution of SCG, it also makes the instrumentation and data acquisition process more expensive and labor-intensive. On the other hand, our vision-based pipeline can simultaneously estimate SCG signals from multiple points on the chest without affecting the computational cost of the data acquisition or analysis. For example, we tested our pipeline to obtain SCG signals from three locations on the sternum (Fig. 9). This feature opens the door for further investigations of the SCG signals and their intrasubject variability at a lower cost compared to the conventional methods.

Furthermore, during the data acquisition and preprocessing, we tried to align the *x* and *y* axes of the video with those of the accelerometers. However, since we simultaneously estimated the SCG signals of three locations along the sternum from the same video, the *y*-axis of the accelerometers placed on the manubrium and xiphoid process may not always be perfectly aligned with the *y*-axis of the camera. This is partly because the chest surface is not flat, and thus those accelerometers’ top surface may not be parallel to the smartphone surface (i.e., not perpendicular to the optical axis of the camera). This can consequently result in estimating SCG$^\mathrm{{v}}$ components in slightly different directions than the SCG$^\mathrm{{g}}$. As a result, the correlation between the two signals can be affected by this misalignment, especially in the time domain.

In this work, we estimated the SCG signals from videos by tracking the motion of QR codes attached to the accelerometers on the chest. However, it is important to note that the accuracy of this method depends on the attachment quality of the QR code to the accelerometer. For example, a poorly attached QR code may compromise the SCG$^\mathrm{{v}}$ results. Moreover, any limitations or obstructions in the camera’s line of sight can adversely affect the performance of our vision-based pipeline. In addition, the basic premise of the template tracking method is that the appearance of the object remains unchanged throughout the video. But in reality, the appearance of an object changes over time, e.g., due to rotation, geometric transformation, or textural alteration. An option for considering these changes is to update the template over time with a new template from the current frame of the video which can be done every frame or alternatively every *m* frames. However, a serious problem in constructing such an adaptive tracking system is that, with every template update, a small error is added to the location of the template. The tracked region eventually drifts away from the actual template location as a result of the accumulation of these errors. Matthews et al.^44^ proposed a solution for this problem by correcting and updating the template using a combination of the reference templates retrieved from the initial and the most recent frames. In this feasibility study, we also used this strategy to correct and update the template by keeping the first template around, i.e., the QR code image extracted from the first frame *QR*(*x*) and utilizing it to correct the drift in $QR_{m+1}({x})$. However, since the chest vibrations caused by the heart’s mechanical activity have a small amplitude, we did not find any significant difference between using the Lucas–Kanade template tracking method with and without a template correction. Thus, we only presented the results without a template correction.

Eventually, the high DTW similarity and the good agreement between the $HR_{SCG}$ and gold-standard HR values reported in this study proved the feasibility of our non-contact SCG measurement method. This study is the first step of our efforts to develop an accurate and robust pipeline to estimate SCG signals from the chest videos under real-life conditions such as capturing the chest videos using the front camera while the smartphone is held by the user (i.e., in the presence of a camera vibration noise). Considering the growing number of smartphone users and the ever-increasing investigations on the utility of SCG for the monitoring and diagnosis of cardiovascular conditions, this novel technique may provide an affordable and widely available method to assess cardiovascular health and refer those users at high risk to medical team for further evaluation of their conditions.

## Conclusions

In this work, we have developed a vision-based pipeline to estimate the SCG signals in the right-to-left and head-to-foot directions from the chest videos recorded using a smartphone. We validated our pipeline by comparing the vision-based SCG signals with the gold-standard signals measured by tri-axial accelerometers. We have demonstrated that a relatively high correlation existed between the gold-standard and vision-based estimations of the SCG signals. Overall, this work demonstrates the possibility of extracting cardiac vibrations from normal phone videos. More studies can lead to a paradigm shift in developing accessible and affordable cardiac remote monitoring tools.

## Data Availability

The data generated and analyzed during the current study are not publicly available due to the IRB requirements but may be available from the corresponding author on a reasonable request.
